# Continuum of care and survival in patients with metastatic colorectal cancer: results of the real-world prospective, longitudinal cohort PROMETCO study

**DOI:** 10.1016/j.esmogo.2025.100214

**Published:** 2025-09-03

**Authors:** M. Koopman, R. Garcia-Carbonero, C. Pinto, A. Mitroshkin, G. Bodoky, L. Mineur, V. Bourgeois, M. Mare, A. Ruiz-Casado, A. Fernandez Montes, J.M. O’Connor, A. Sullivan, E. Choucair, B. Chevallier, F. Marti Marti, J.-B. Bachet

**Affiliations:** 1 Department of Medical Oncology, University Medical Centre Utrecht, Utrecht University, Utrecht, The Netherlands; 2 Oncology Department, Hospital Universitario Doce de Octubre, Imas12, Facultad y Departamento de Medicina, Universidad Complutense de Madrid (UCM), Madrid, Spain; 3 Medical Oncology, Comprehensive Cancer Centre, AUSL – IRCCS di Reggio Emilia – Reggio Emilia, Italy; 4 Klinikum Freudenstadt, Akademisches Lehrkrankenhaus der Universität Tübingen, Freudenstadt, Germany; 5 Dél-Pesti Centrumkórház, Szent László Telephely, Budapest, Hungary; 6 GI and Liver Oncology Unit, Institut of Cancer Sainte Catherine Avignon Provence, Avignon, France; 7 Boulogne-sur-Mer Hospital Center, Rue Jacques Monod, Boulogne-sur-Mer, France; 8 Medical Oncology Unit, Mediterranean Institute of Oncology, Catania, Italy; 9 Department of Medical Oncology, Hospital Universitario Puerta de Hierro, Majadahonda, IDIPHISA, Madrid, Spain; 10 Complexo Hospitalario Universitario de Ourense, Calle Ramon Puga Noguerol, Orense, Spain; 11 Instituto Alexander Fleming, Buenos Aires, Argentina; 12 Servier Pharmaceuticals, Boston, USA; 13 Servier, Suresnes, France; 14 Department of Medical Oncology, The Christie NHS Foundation Trust, Manchester, UK; 15 Sorbonne Université, Service d’hépato-Gastro-Entérologie, Groupe Hospitalier Pitié Salpêtrière, APHP, Paris, France

**Keywords:** mCRC, third-line, real-world data, overall survival

## Abstract

**Background:**

PROMETCO is the first international, prospective study investigating the continuum of care, including prescribing patterns, efficacy and safety in patients with metastatic colorectal cancer (mCRC) at later therapy lines in a real-world setting.

**Materials and methods:**

Adults with mCRC and two disease progressions since diagnosis of mCRC who were willing to receive subsequent treatment and gave informed consent were included. The study consisted of retrospective medical chart data collection pre-inclusion and a prospective observational period post-inclusion. Endpoint data presented include patient characteristics, treatment patterns and efficacy including progression-free survival (PFS) per treatment line and overall survival (OS).

**Results:**

As of July 2023, 738 mCRC patients from 96 centres in 18 countries were recruited. 48.9% of patients had *RAS*-mutated and 5.0% *BRAF*-mutated mCRC. Between mCRC diagnosis and death or withdrawal, patients were frequently exposed to fluoropyrimidine (99.0%), irinotecan (96.2%), oxaliplatin (88.4%), anti-vascular endothelial growth factor (78.7%) and anti-epidermal growth factor receptor (40.1%). Median OS was 36.4, 7.1, and 6.6 months from mCRC diagnosis, inclusion into PROMETCO and third-line (3L) treatment initiation, respectively. Median PFS decreased significantly from first-line (9.2 months) to 3L (2.7 months) and remained consistent from 3L to sixth-line treatment (∼2.3 months). Median OS from diagnosis was 32.7, 26.8, and 40.6 months in *RAS*-mutated, *BRAF*-mutated, and *RAS/BRAF* wildtype mCRC patients, respectively.

**Conclusions:**

PROMETCO provided information on real-world prescribing patterns and efficacy. OS from mCRC diagnosis and PFS from 3L and beyond were similar to previous long-term follow-up data from clinical trials.

## Highlights

• PROMETCO investigated treatment patterns throughout the continuum of care in metastatic colorectal cancer.
• Treatment exposure in the 738 patients was largely as expected if guidelines were adhered to.
• Median OS from diagnosis and 3L treatment initiation was 36.4 months and 6.6 months, respectively.
• Median PFS decreased from 1L to 3L, then was consistent up to and including sixth-line.
• Survival results were similar to previous long-term follow-up data from clinical trials.

## Introduction

The primary treatment goal for patients with unresectable metastatic colorectal cancer (mCRC) is symptom control and prolongation of survival with preservation or improvement of quality of life.^1^ According to international guidelines, several regimens and treatments are recommended according to disease site and extent, resectability, the presence of microsatellite stability (MSS) or instability (MSI), the patient’s tumour *RAS*/*BRAF* mutation status, clinical presentation, age, performance status and comorbidities.^2^^,^^3^ In fit patients, treatments include chemotherapy doublet regimens with or without bevacizumab or epidermal growth factor receptor (EGFR) inhibitors (cetuximab, panitumumab), or triplet regimens with or without bevacizumab, and pembrolizumab for patients in the deficient mismatch repair (dMMR)/MSI subgroup. Recommended first-line (1L) therapy for mCRC patients who are unfit for intensive doublet or triplet chemotherapy is fluoropyrimidine-based chemotherapy with or without bevacizumab.^2^ Second-line (2L) treatments include alternative chemotherapy regimens to those used in the 1L regimen, with or without angiogenesis inhibitors (bevacizumab, aflibercept, ramucirumab) or EGFR inhibitors (according to molecular status and tumour sidedness), or alternatives such as encorafenib plus cetuximab in patients with *BRAF* V600E-mutated mCRC and ipilimumab plus nivolumab in patients with dMMR/MSI.^2^ Trifluridine*/*tipiracil (FTD/TPI) with or without bevacizumab, regorafenib and fruquintinib, are approved therapies for third-line (3L) therapy or beyond, indicated for patients who have progressed through all standard therapies.^2^, ^3^, ^4^, ^5^, ^6^, ^7^ Furthermore, recent studies in 3L or later lines showed efficacy of targeted therapies in molecular selected patients: anti-HER2 combinations in HER2-positive tumours;^8^^,^^9^ and *KRAS* inhibitors in *KRAS* G12C-mutated tumours.^10^^,^^11^

In the 3L setting, good prognosis is predicted by low tumour burden (less than three metastatic sites at 3L therapy initiation) and less aggressive and/or more chemosensitive disease (≥18 months from diagnosis of first metastasis to start of 3L therapy).^12^ In general, advances in mCRC treatment have now improved median overall survival (OS) up to 30 months (and >40 months in selected subgroups of patients) in clinical trials, through incremental increases in OS throughout the continuum of care,^2^ and data on combination 3L treatment such as FTD/TPI plus bevacizumab and fourth-line treatment such as fruquintinib suggest that this OS can be prolonged further.^6^^,^^7^

mCRC treatment has changed rapidly in recent years and as a result, patients may receive 1L or 2L agents in clinical practice (e.g., pembrolizumab, nivolumab-ipilimumab, encorafenib + cetuximab) that were not available when the phase III studies for later-line treatments were conducted. Additionally, treatment availability varies between countries according to reimbursement of treatments and accessibility to molecular testing. Treatment recommendations are based on selected, mostly younger and fitter, patients included in randomised controlled trials, but real-world studies can provide invaluable information on actual prescribing patterns, efficacy and safety in a broader population. Several studies have investigated real-world treatment of mCRC in later lines, however they recruited patients treated with specific agents or were limited to a single country.^13^, ^14^, ^15^, ^16^, ^17^, ^18^ PROMETCO (NCT03935763) is the first international, prospective real-world study to investigate treatment patterns throughout the continuum of care. The aim is to report treatment patterns and outcomes including OS and progression-free survival (PFS) per treatment line in patients with two disease progressions from diagnosis of advanced mCRC, to help inform clinical practice.

## Methods

### Study design

PROMETCO is a prospective, longitudinal, observational cohort study collecting data on patients with mCRC who had two disease progressions since the first diagnosis of metastatic disease, are willing to receive subsequent treatment, and gave informed consent. The protocol was published and contains further detail on the methodology.^19^ The study aimed to recruit patients at 125 centres in 18 countries (Argentina, Austria, Belgium, Croatia, Czech Republic, France, Germany, Greece, Hungary, Ireland, Italy, The Netherlands, Portugal, Slovenia, Spain, Sweden, Switzerland and the UK). Academic or community-based medical centres in the selected countries of interest that were experienced in the treatment and management of CRC and in conducting observational studies were approached for potential participation in the study. Patient recruitment began in March 2019, ended in October 2022, and the study was completed in April 2024.

The study was conducted according to the principles of the Declaration of Helsinki, the Guidelines for Good Pharmacoepidemiology Practices of the International Society for Pharmacoepidemiology 2016 and the Strengthening the Reporting of Observational Studies in Epidemiology guidelines. The study protocol was approved by the ethics committees of participating centres. All patients must provide written informed consent, the documents of which were reviewed by the patient advocacy group Digestive Cancer Europe before enrolment commenced. To limit bias in the selection of patients, investigators enrolled all consecutive patients providing consent and meeting the selection criteria, regardless of treatment, health status, or other considerations. Source data verification was conducted at each centre (minimum one per centre, and additionally as needed) to limit information bias for both prospective and retrospective data. During the source data verification, medical records data were compared with the information provided in the study database for a randomly selected patient. The collection of baseline information helped to identify the presence of elevated background risk, so that event rates could be compared through subgroup analysis to determine whether the elevated risk was likely related to background risk or the use of different treatments.

### Participants

Inclusion criteria were: patients aged ≥18 years, confirmed diagnosis of mCRC, two disease progressions since the first diagnosis of metastasis that led to the first systemic treatment, and willingness to receive subsequent treatment. All eligible patients were recruited, with no maximum threshold. Patients were excluded if they were receiving treatment for another cancer, participating in an investigational clinical trial or did not have the ability or mental capacity to participate. Treatment decisions were made by each patient’s treating physician according to local standard medical practice.

### Assessment

The study consisted of retrospective medical chart data collection before inclusion and a prospective observational period after inclusion. At enrolment, data on patient and clinical characteristics, treatment from mCRC diagnosis, medical history, and concomitant diseases were obtained retrospectively through a medical chart review using electronic case report forms (eCRFs) and the ClinInfo electronic data capture system. Information on data collected can be found in Supplementary Table S1, available at https://doi.org/10.1016/j.esmogo.2025.100214. Prospective data were collected using the same eCRFs in conjunction with regularly scheduled follow-up visits according to local standard medical practices for up to 18 months from study entry. There was no fixed schedule for the follow-up visits. Patients were considered to have completed the study if they were lost to follow-up, withdrew from the study, or died. To inform survival, the date of death was captured for those patients who were alive after 18 months of follow-up.

### Outcomes

Effectiveness outcomes included response to treatment (response, disease progression or stable disease assessed using the local medical standard assessments; objective response rate was the number of patients with complete or partial response, disease control rate was the number of patients with complete or partial response, or stable disease), date of response, OS and PFS calculated in days from baseline visit plus one day. OS and PFS were assessed from inclusion in PROMETCO, from mCRC diagnosis, and from start of 3L treatment (defined as the treatment given when the first and second treatment do not work or stop working) until outcome (death from any cause for OS; progression or death due to any cause for PFS) or until end of 18-month follow up. PFS was also assessed from all lines of therapy (LOTs), up until seventh-line. A start of a new LOT was defined as the first administration of a new cytotoxic agent and/or a new targeted therapy.

Outcomes were analysed in the whole population, and in the following subgroups: specific molecular subgroups (*RAS/BRAF* status and MSS/MSI status); and according to the prognostic subgroups defined by Tabernero et al.^12^ Good prognosis characteristics (GPC) were less than three metastatic sites at study entry (low tumour burden) and ≥18 months from diagnosis of metastatic disease to study entry (indolent disease). Best prognosis characteristics (BPC) was the subgroup of the GPC patient group who also had no liver metastasis. The remaining patients were considered to have poor prognosis characteristics (PPC).

### Statistical methods

The primary effectiveness outcome was OS and the secondary outcome measure was PFS per treatment line. Descriptive statistics were used for most outcome measures, and continuous variables were summarized using mean, standard deviation, median and range. Categorical variables were reported as the number and percentage of patients. PFS and OS were summarized using Kaplan–Meier survival analysis. Quality control and data validation programming (i.e., distance training of study site personnel, and on-site and remote monitoring) was applied to ensure the consistency and accuracy of the statistical analysis datasets, statistical analyses, and their associated output.

## Results

### Patient population

Baseline characteristics from 738 mCRC patients from 96 centres in 18 countries were collected (Table 1; Supplementary Figure S1, available at https://doi.org/10.1016/j.esmogo.2025.100214). Of 738 patients, 361 had *RAS*-mutated mCRC (48.9%), 37 had *BRAF*-mutated mCRC (5.0%), 236 (32.0%) had *RAS/BRAF* wildtype mCRC, 9 (1.2%) had both *RAS* and *BRAF*-mutated mCRC, and *RAS* and *BRAF* mutation status was unknown for 57 (7.7%) patients. Baseline characteristics by *RAS/BRAF* mutational status is shown in Supplementary Table S2, available at https://doi.org/10.1016/j.esmogo.2025.100214. Twelve (1.6%) patients had MSI-high mCRC, 431 (58.4%) patients had MSS mCRC, and 295 (40.0%) patients had an unknown MSI/MSS status. Baseline characteristics data by MSI status is available in Supplementary Table S3, available at https://doi.org/10.1016/j.esmogo.2025.100214. In total, 442 (60.3%) patients fulfilled the criteria for GPC, and of these, 130 (17.7%) fulfilled the criteria for BPC. The remaining 291 (39.7%) fulfilled the criteria for PPC. Patient status as of July 2023 is shown in Supplementary Figure S2, available at https://doi.org/10.1016/j.esmogo.2025.100214.

**Table 1:** Baseline patient characteristics

Baseline characteristic		Patients (*N* = 738)
Age, years	Median (min, max)	68.0 (31.0, 87.0)
Sex, *n* (%)^a^		
	Female	302 (41.1)
	Male	433 (58.9)
ECOG PS^b^, *n* (%)		
	0	283 (39.5)
	1	378 (52.8)
	2	51 (7.1)
	3	4 (0.6)
	ND	22
Time between mCRC diagnosis and PROMETCO inclusion (months)^a^	Median (min, max)	27 (3, 215)
Time on treatment before PROMETCO inclusion (months)^a^	Median (min, max)	13.2 (0.5, 101.6)
Number of metastatic sites, *n* (%)^c^		
	<3	659 (89.8)
	≥3	75 (10.2)
Type of metastasis, *n* (%)^a^		
	Synchronous	480 (65.3)
	Metachronous	255 (34.7)
Tumour primary site, *n* (%)^c^		
	Right (cecum + ascending colon/transverse colon)	207 (28.2)
	Left (descending colon/sigmoid colon)	311 (42.4)
	Rectum	262 (35.7)
*RAS/BRAF* status, *n* (%)		
	*RAS* mut^d^	361 (48.9)
	*BRAF* mut^d^	37 (5.0)
	*RAS/BRAF* WT	236 (32.0)
	*RAS/BRAF* mut	9 (1.2)
	Unknown^e^	57 (7.7)
MSI/MSS status, *n* (%)		
	MSI high	12 (1.6)
	MSS	431 (58.4)
	Unknown	295 (40.0)
Prognosis group, *n* (%)^f^		
	Poor	291 (39.7)
	Good	442 (60.3)
	Best	130 (17.7)
Distribution of metastatic sites, *n* (%)		
	Liver	541 (73.3)
	Lung	285 (38.6)
	Peritoneal carcinomatosis	105 (14.2)
	Bone	29 (3.9)
	Adrenal gland	22 (3.0)
	Other^g^	159 (1.4)

ECOG PS, Eastern Cooperative Oncology Group performance status; max, maximum; mCRC, metastatic colorectal cancer; min, minimum; MSI, microsatellite instability; MSS, microsatellite stable; mut, mutant; ND, not determined; WT, wildtype.

a *n* = 735 due to missing data.

b Percentage based on n observed per group (i.e. not including ND values; *n* = 716).

c *n* = 734 due to missing data.

d *RAS* mutant and *BRAF* mutant patient numbers excludes *RAS/BRAF* mutant patients.

e Unknown only includes patients with unknown *BRAF* and *RAS* status [excludes patients with *BRAF* wildtype/unknown *RAS* status (*n* = 6), and patients with *RAS* wildtype/unknown *BRAF* status (*n* = 29)].

f According to subgroups proposed by Tabernero et al.^12^ ‘Good’ prognosis, defined as less than three metastatic sites at study entry (low tumour burden) and ≥18 months from diagnosis of metastatic disease to study entry (indolent disease); ‘Poor’ prognosis, the remaining patients were considered to have PPC; ‘Best’ prognosis, those in the good prognosis group who had no liver metastasis.

g Brain and skin metastases included in ‘other’.

### Treatment

Median treatment duration before PROMETCO inclusion was 13.2 months (minimum 0.5 months, maximum 101.6 months), while median time between mCRC diagnosis and inclusion was 22.3 months (minimum 3.4 months, maximum 214.9 months). Median time between mCRC diagnosis and inclusion was 20.5 months (minimum 4.9 months, maximum 214.9 months) for *RAS*-mutated patients, 13.5 months (minimum 3.4 months, maximum 48.5 months) for *BRAF*-mutated patients, and 24.2 months (minimum 5.6 months, maximum 140.7 months) for *RAS/BRAF* wildtype patients. Median time between mCRC diagnosis and inclusion was 38.8 months, 30.8 months, and 24.4 months for patients who had at least one lung, liver or colorectal surgery, respectively. Patients were exposed to fluoropyrimidine (99.0%), irinotecan (96.2%), oxaliplatin (88.4%), anti-vascular endothelial growth factor (VEGF) therapy (78.7%), FTD/TPI (75.2%), anti-EGFR (40.1%), and regorafenib (25.3%) between mCRC diagnosis until death or withdrawal from the study. From mCRC diagnosis to death, 64.6% of the patients had colorectal surgery, 5.0% had lung surgery, and 22.9% had liver surgery. Analysis of mCRC treatments by *RAS*/*BRAF* mutation status is shown in Supplementary Table S4, available at https://doi.org/10.1016/j.esmogo.2025.100214.

### Effectiveness

**Figure 1:**
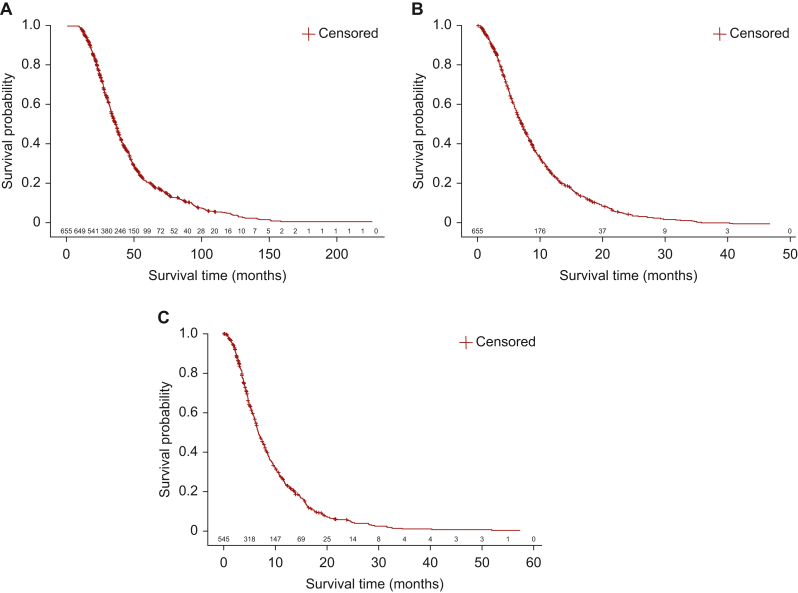
**Kaplan–Meier curves of OS in patients with mCRC.** (A) from diagnosis to the end of the study; (B) from second disease progression and inclusion into PROMETCO to the end of the study; and (C) from third treatment line to the end of the study.

**Figure 2:**
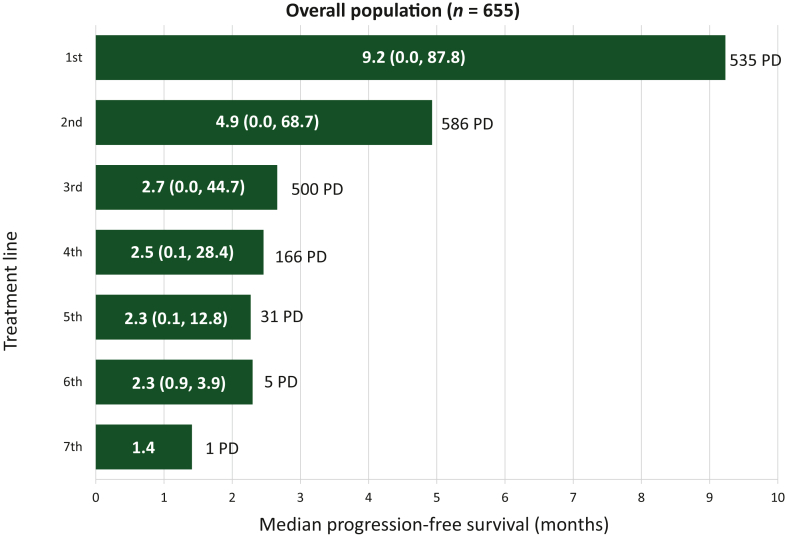
**Median (min, max) PFS in patients at each line of treatment**. Progression-free survival (PFS) definition: start, start date of treatment line; end, event (date of PD)/event (date of death)/censor (end of line of treatment)/censor (end of line of treatment)/censor (end of last available day). max, maximum; min, minimum; PD, events of progressive disease.

At this data cut-off of 1 July 2023, 655/738 of the mCRC patients enrolled in PROMETCO had completed the study and were included in the survival analyses, and the study is still ongoing. The median OS from mCRC diagnosis to the end of the study was 36.4 months [95% confidence interval (CI): 33.9-38.3 months; Figure 1A]. The median OS from inclusion into PROMETCO, i.e. after second disease progression, to the end of the study was 7.1 months (95% CI: 6.5-7.6 months; Figure 1B). The median OS from 3L treatment initiation to the last registered follow-up was 6.6 months (95% CI: 6.2-7.4 months; Figure 1C). As expected, the median PFS decreased significantly from 1L [9.2 months (95% CI: 8.6-10.2 months)] to seventh-line treatment [1.4 months (95% CI: not calculable); Figure 2]. The median PFS was similar at all LOTs between 3L and sixth-line treatment, ranging between 2.3 and 2.7 months.

### The effect of RAS/BRAF mutations on survival

**Figure 3:**
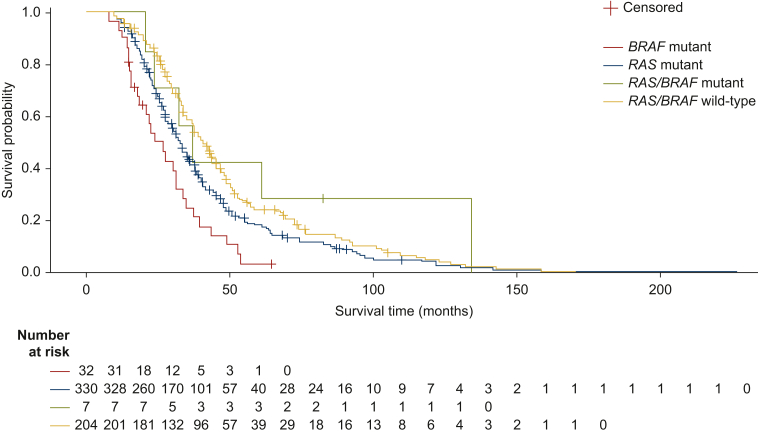
OS in patients with RAS or BRAF-mutated mCRC from mCRC diagnosis to the end of the study^a^

**Figure 4:**
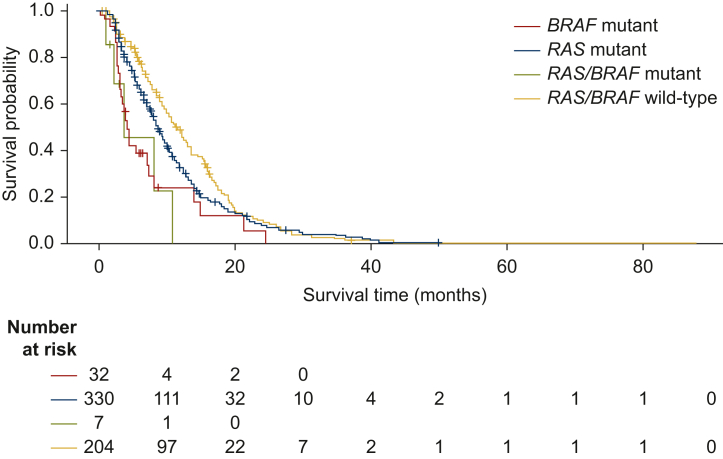
PFS in first-line in patients with RAS or BRAF-mutated mCRC.^a^

When survival from mCRC diagnosis to the end of the study was analysed by molecular profile at inclusion, it was observed that *RAS* and *BRAF* biomarkers have prognostic value. Survival was shortest for patients with *BRAF*-mutated mCRC [26.8 months (95% CI: 18.1-31.0 months)], while *RAS/BRAF* wildtype appeared to be associated with the longest median OS [40.6 months (95% CI: 37.2-45.5 months); Figure 3]. PFS at 1L was shortest for patients with *BRAF*-mutated mCRC [4.0 months (95% CI: 3.0-7.2 months)] and *RAS/BRAF* wildtype appeared to be associated with the longest median 1L PFS [11.6 months (95% CI: 9.7-12.9 months); Figure 4].

When OS after starting 3L therapy was analysed by molecular profile, it was observed that prognostic impact of *RAS* and *BRAF* mutations in later lines was less evident. Median OS was 6.4 months [95% CI: 5.9-7.4 months], 7.3 months [95% CI: 4.7-8.2 months], and 7.0 months [95% CI: 6.0-9.2 months] in patients with *RAS*-mutated mCRC, *BRAF*-mutated mCRC, and *RAS/BRAF* wildtype mCRC, respectively. Two patients with *BRAF*-mutated mCRC were also MSI-high.

### The effect of MSS status on survival

Median OS from mCRC diagnosis to study end for MSI-high patients (*n* = 9) was 32.5 months [95% CI: 16.7-35.2 months], and for MSS patients (*n* = 355) was 34.1 months [95% CI: 32.4-37.4 months; Supplementary Figure S3, available at https://doi.org/10.1016/j.esmogo.2025.100214].

### The effect of good versus poor prognosis classifications on survival

**Figure 5:**
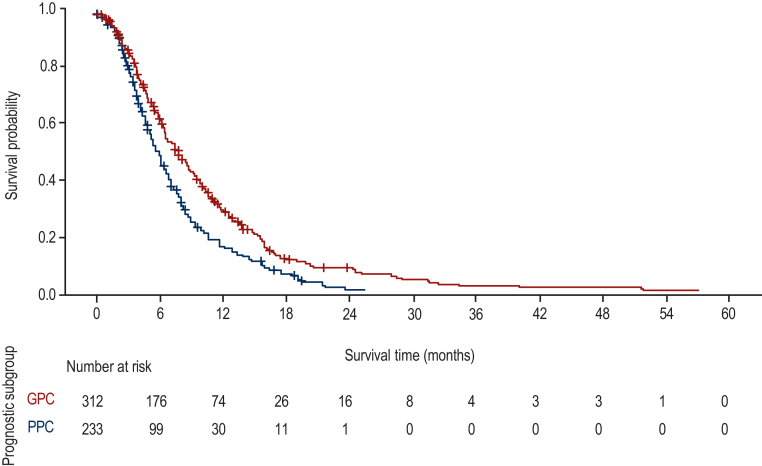
OS from 3L treatment by prognostic subgroup.

When survival was analysed from 3L treatment initiation to the end of the study according to prognosis classification status, as expected, median OS was longest in the BPC group [9.5 months (95% CI: 7.3-11.6 months)], and shortest in the PPC group [5.9 months (95% CI: 5.1-6.6 months); Figure 5]. The median OS in patients with GPC was 7.9 months [95% CI: 6.6-8.7 months; Figure 5]. As expected, PFS at 1L was longest in the BPC group [14.5 months (95% CI: 11.1-16.2 months)], and shortest in the PPC group [5.7 months (95% CI: 5.2-6.4 months)]. Median PFS in patients with GPC was 13.6 months [95% CI: 12.7-14.7 months].

## Discussion

PROMETCO aimed to provide invaluable information on actual prescribing patterns and efficacy in mCRC patients in a broad, real-world population. Survival from mCRC diagnosis in the real-world was similar to previous long-term follow-up data from clinical trials of 1L therapy, whereas survival from 3L therapy initiation was not as long as in recent clinical trials of 3L therapy.

In this well-defined cohort of patients in PROMETCO, median OS from diagnosis was 36.4 months, which is longer than previously reported survival in clinical trials [∼30 months (in phase III trials and in large observational series or registries)].^2^ This is likely due to the selective population included in PROMETCO that required patients to have at least two disease progressions and hence excluded patients who did not survive long enough to reach 3L therapy. Previous studies have shown conditional survival in mCRC patients who underwent surgical resection where probability of achieving 5-year OS for CRC increased gradually with additional time survived.^20^ An example of how this may manifest in the current data is shown by the median OS data in *BRAF*-mutated mCRC patients. As expected, patients with *BRAF*-mutated mCRC had a shorter median OS from mCRC diagnosis than patients with *RAS*/*BRAF* wildtype mCRC (26.8 versus 37.1 months, respectively). However, the prognostic impact of having *BRAF*-mutated mCRC was not found for OS after starting 3L therapy, probably because of a selection effect under 1L and 2L. This notable finding of a loss of prognostic significance for *BRAF* mutations from 3L treatment has implications for future trials of later-line treatment, in which stratification by *BRAF* mutation status may not be required. However, information on *BRAF* mutation sub-type was not available in PROMETCO, and the possible impact of the prevalence of the *BRAF* V600 sub-type, which is known to confer a particularly poor prognosis,^21^ cannot be assessed. Only 5.0% of the population in PROMETCO had *BRAF*-mutated mCRC and this small sample size may also make it problematic to draw conclusions on the prognostic significance of *BRAF* mutation on OS.

The median OS from mCRC diagnosis to the end of the study was similar for MSS patients (34.1 months) and MSI-high patients (32.5 months). However, with the high proportion of patients with missing information on MSI/MSS status (40.0%), and as the proportion of MSI-high patients was so low (1.6%), analysis was limited in this population. MSI-high patients are recommended a different treatment pathway (which includes the addition of immunotherapies such as pembrolizumab into 1L regimens), and so the high proportion of patients with unknown MSI/MSS status in this study is surprising. Furthermore, in Europe, which represents most of the countries in this study, access to pembrolizumab in 1L therapy and ipilimumab/nivolumab after 1L was not possible until January 2021. Since recruitment for PROMETCO began in March 2019, a proportion of the population may not have been tested as rigorously for MSI/MSS status. Because of this, the high proportion of patients with missing MSI/MSS status may not have had a large impact on the treatment patterns and outcomes data collected in PROMETCO. Approximately 4%-5% of patients with mCRC are MSI-high,^22^ and many of these patients will receive immunotherapy and have a durable response that may mean they do not have two disease progressions. The low proportion of MSI-high patients in PROMETCO is therefore not surprising. However, more real-world data on the effectiveness of 3L and further treatment in patients with MSI-high mCRC will be available from future studies.

The median OS after starting 3L therapy was 6.6 months in PROMETCO. The median age in PROMETCO (67 years) was higher than that observed in the 3L studies, RECOURSE and SUNLIGHT (63 years).^4^^,^^6^ This higher median age reflects current clinical practice. The PROMETCO population may also have been frailer than those in clinical trials – for example, exclusion criteria in both RECOURSE and SUNLIGHT included inadequate organ function and ECOG PS score >1. Observational studies of 3L treatment with FTD/TPI or regorafenib have also recruited younger populations than the PROMETCO population (median 62 years^13^ and 65 years,^16^ respectively), but there is no clear explanation for this difference. Although of lower relevance given the loss of prognostic value from 3L treatment onwards, the proportion of patients with *RAS* and/or *BRAF-*mutated mCRC in PROMETCO was in line with those observed in both the observational studies of 3L treatment PRECONNECT^13^ and CORRELATE,^16^ and in the randomized 3L clinical trial SUNLIGHT.^6^

Recent clinical trial data suggest that further improvements in OS compared with those observed in PROMETCO are possible with 3L combination therapy. In SUNLIGHT, the median OS from 3L initiation with the combination of FTD/TPI and bevacizumab was 10.8 months versus 7.3 months for the FTD/TPI alone.^6^ A very small proportion of patients (1%) received this combination in the PROMETCO study. Recruitment for PROMETCO ended in October 2022, and the results from the SUNLIGHT trial were not reported until January 2023, which explains the infrequent use of FTD/TPI + bevacizumab. As the proportion of patients receiving FTD/TPI + bevacizumab was small, it could be misleading to report effectiveness outcomes in this population at this time. If further follow-ups on the PROMETCO study include more patients who received FTD/TPI + bevacizumab as 3L therapy, outcomes will be reported.

The median OS after starting 3L was shorter than the median OS from inclusion in PROMETCO, reflecting the time difference between second progression and 3L treatment initiation. PPC was associated with worse OS from 3L treatment (5.9 months) than GPC (7.9 months) or BPC (9.5 months). In the RECOURSE study, median OS was higher in the GPC subgroup (9.3 months), and lower in the PPC subgroup (5.3 months) for patients treated with FTD/TPI compared with the broader population in PROMETCO. While BPC was not analysed in the RECOURSE study, the higher OS observed in the GPC group could be a result of the higher proportion of patients with no liver metastasis in the GPC group of the FTD/TPI arm of RECOURSE (97/261 [37.2%]) compared with PROMETCO (130/442 [29.4%]). The proportion of patients in the GPC group was higher in PROMETCO (60.3%) than in the RECOURSE study (48%).^12^

Median total duration under treatment before PROMETCO inclusion was 13.2 months, and median time between mCRC diagnosis and inclusion was 22.3 months, a difference that may suggest the use of treatment breaks in the real world. Previous studies have shown that the use of managed treatment breaks does not have a detrimental effect on OS in mCRC.^23^ However, treatment breaks in PROMETCO are likely due to surgery of primary tumours or metastases, as median time between mCRC diagnosis and inclusion was particularly long (24.4-38.8 months) in patients receiving surgery (lung, liver, or colorectal). Patients with *BRAF*-mutated mCRC had a shorter time between mCRC diagnosis and inclusion (14 months) than the overall population. As the *BRAF* mutation is a marker of poor prognosis it is possible that these patients progress though LOTs quicker and are therefore eligible for PROMETCO inclusion earlier than the overall population;^21^ however, as these patients would need to be fit for 3L treatment, this suggestion may not fully explain the shorter time from diagnosis in this patient subgroup.

mCRC treatment guidelines are based on randomised clinical trials,^2^ which are potentially limited by the inclusion of selected patients only. Furthermore, physicians’ previous experience or beliefs are likely to affect their treatment decisions, causing actual prescribing patterns to differ from evidence-based recommendations, and resulting in a lack of clarity regarding how recommended agents should be used in clinical practice. PROMETCO is a real-world study and as such can examine treatment variability in clinical practice and provide an accurate picture of real-world practice and outcomes in a range of countries. During the disease course, between diagnosis and end of the study, patients were most frequently exposed to fluoropyrimidine (98.5%), which suggests treatment is mostly consistent with current European Society of Medical Oncology guidelines.^2^^,^^4^^,^^13^ The high proportion of patients (75%) receiving FTD/TPI also indicates that real-world practices have evolved alongside the guidelines. Real-world studies also often include patients who have poor performance status or comorbid conditions, which can lead to their exclusion from randomized, controlled trials. A strength of the PROMETCO study is that the exclusion criteria are minimal and non-stringent. Another strength of the trial is that it is multicentre and multinational, however it is a primarily European study with ∼97% of patients coming from Europe.

The main limitations of PROMETCO are those common to observational studies. One limitation of observational studies is that data may not be collected as stringently as in clinical trials, but there was limited missing data in this study. However, information was not available for patients that did not receive treatment after two progressions of disease, which therefore prevents any assessment of systematic differences between included and excluded populations or of selection bias in the included population. The study is also subject to bias and confounding factors, particularly in the collection of retrospective data; however, the authors aimed to overcome some of these limitations by collecting data prospectively and retrospectively.

## Conclusion

In conclusion, PROMETCO gathered a large amount of data on actual prescribing patterns, efficacy and safety in a well-defined, real-world cohort of patients who had two progressions of disease and were possibly older and frailer than clinical trial populations. In total, 48.9% of patients had *RAS-*mutated mCRC and 5.0% *BRAF*-mutated mCRC. Median OS was 36.4 months, 7.1 months, and 6.6 months from mCRC diagnosis, from inclusion into PROMETCO and from 3L treatment initiation, respectively. The similar PFS in 3L to subsequent LOTs was of particular interest and there was no prognostic value of *BRAF*/*RAS*/tumour sidedness at later lines. Recent clinical trial data suggest that further improvements in OS compared with those observed in PROMETCO are possible with 3L combination therapy or fourth-line treatment.^6^^,^^7^
