# Supplementary figures and images for: Continuum of care and survival in patients with metastatic colorectal cancer: results of the real-world prospective, longitudinal cohort PROMETCO study
Source: ESMO Gastrointest Oncol. 2025 Sep 3;9:100214. doi: 10.1016/j.esmogo.2025.100214 (PMC12836624; doi:10.1016/j.esmogo.2025.100214)

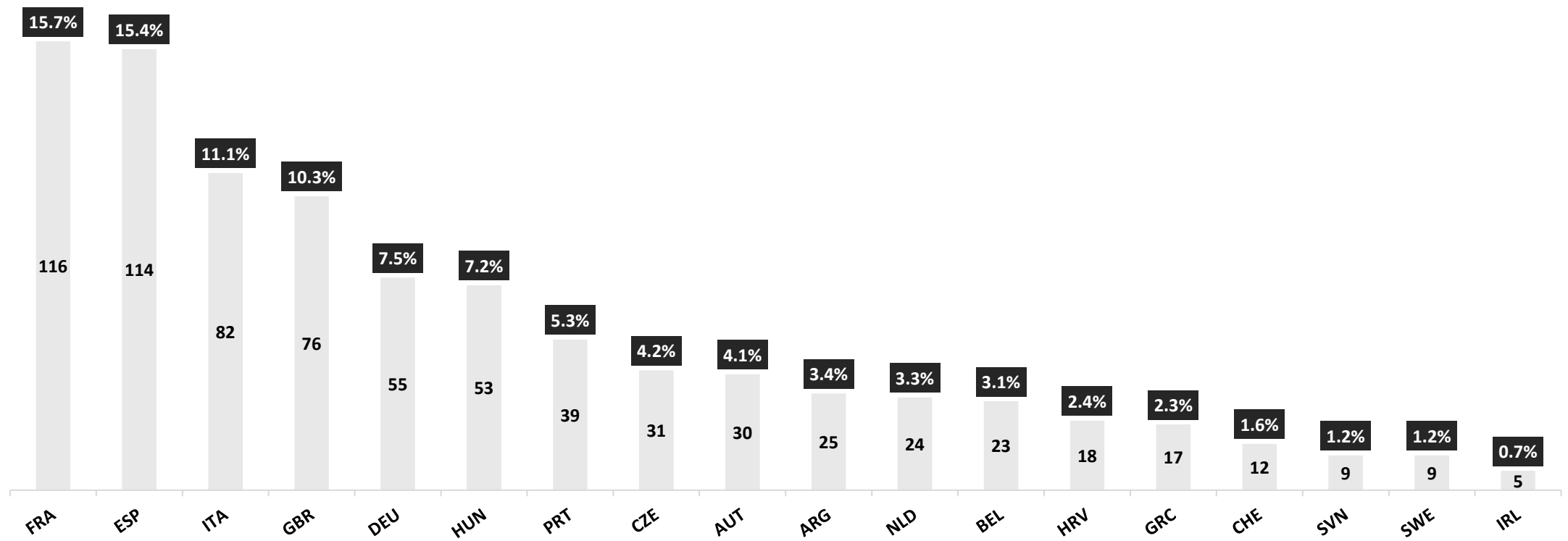

Supplement: Supplementary Figure 1 [file mmc1.pdf]

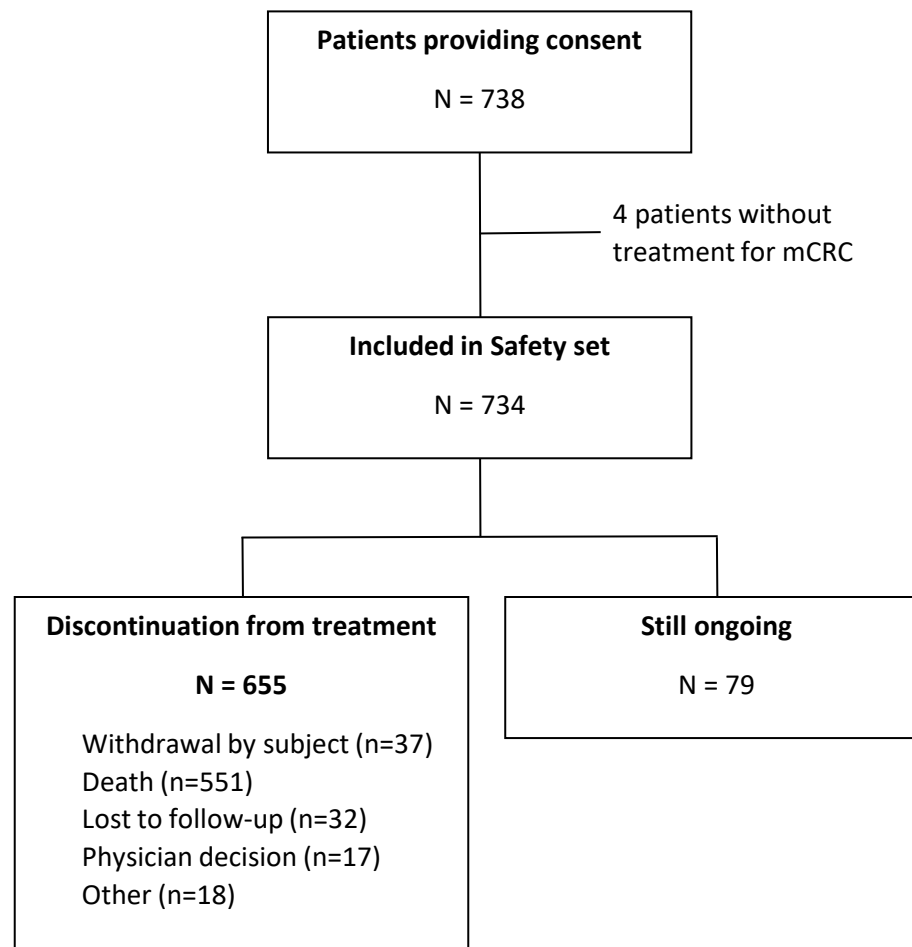

Supplement: Supplementary Figure 2 [file mmc2.pdf]

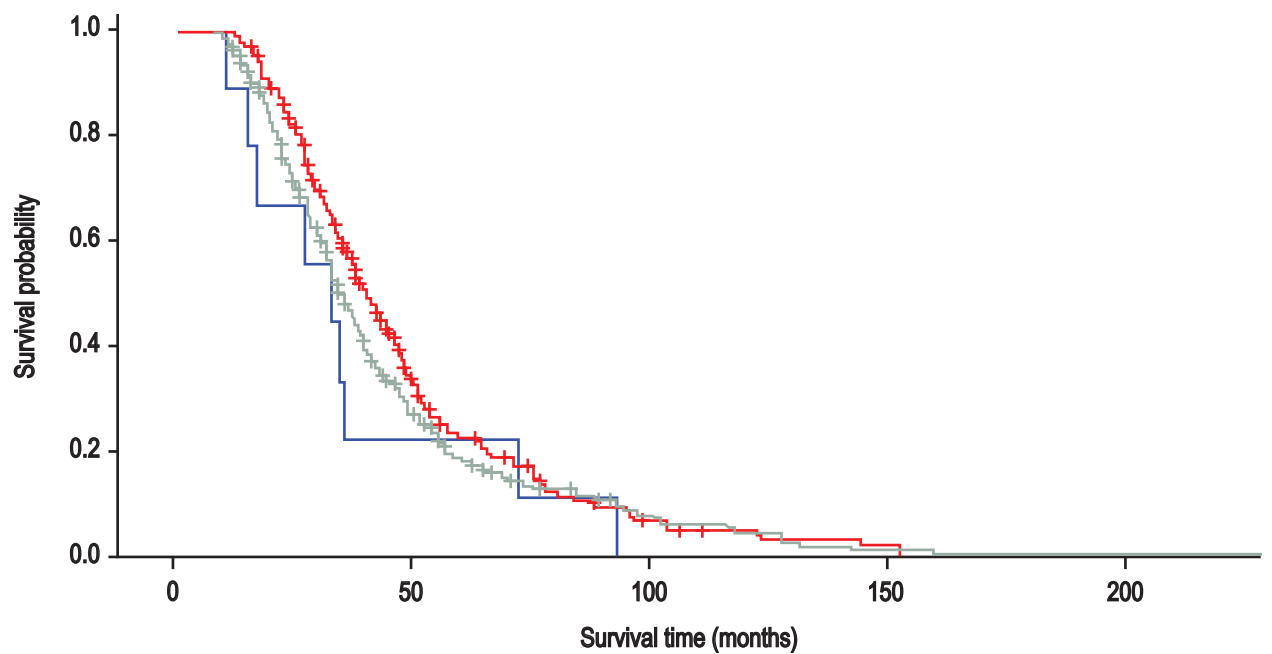

Number at risk

|          |     |     |     |     |     |    |    |    |    |    |    |    |    |   |   |   |   |   |   |   |   |   |   |
|----------|-----|-----|-----|-----|-----|----|----|----|----|----|----|----|----|---|---|---|---|---|---|---|---|---|---|
| MSI-high | 9   | 9   | 6   | 5   | 2   | 2  | 2  | 2  | 1  | 1  | 0  |    |    |   |   |   |   |   |   |   |   |   |   |
| MSS      | 355 | 350 | 282 | 197 | 123 | 80 | 51 | 36 | 30 | 23 | 17 | 13 | 10 | 6 | 4 | 3 | 2 | 2 | 1 | 1 | 1 | 1 | 0 |
| Unknown  | 267 | 266 | 234 | 169 | 114 | 63 | 41 | 30 | 18 | 14 | 9  | 6  | 5  | 3 | 3 | 2 | 0 |   |   |   |   |   |   |

Supplement: Supplementary Figure 3 [file mmc3.pdf]
