# Supplementary material for: Continuum of care and survival in patients with metastatic colorectal cancer: results of the real-world prospective, longitudinal cohort PROMETCO study
Source: ESMO Gastrointest Oncol. 2025 Sep 3;9:100214. doi: 10.1016/j.esmogo.2025.100214 (PMC12836624; doi:10.1016/j.esmogo.2025.100214)
Supplement: Supplementary Material [file mmc4.docx]

**Supplementary File**

**Supplementary Figure 1: Patient distribution by country in the overall study population**

ARG, Argentina; AUT, Austria; BEL, Belgium; CHE, Switzerland; CZE, Czech Republic; DEU, Germany; ESP, Spain; FRA, France; GBR, United Kingdom; GRC, Greece; HRV, Croatia; HUN, Hungary; IRL, Ireland; ITA, Italy; NLD, Netherlands; PRT, Portugal; SWE, Sweden; SVN, Slovenia.

**Supplementary Figure 2: Disposition of patients as of July 2023**

Abbreviations: mCRC, metastatic colorectal cancer.

**Supplementary Figure 3: OS in patients with mCRC from diagnosis to the end of the study in the MSS/MSI subgroups***

| **OS** | **MSI-high (N=9)** | **MSS (N=355)** |
| --- | --- | --- |
| **Event, n (%)** | 9 | 307 |
| **Censored, n (%)** | 0 | 48 |
| **Median (95% CI), months** | 32.5 (16.7-35.2) | 34.1 (32.4-37.4) |
| **Min, max, months** | 10.5, 92.0 | 7.8, 226.9 |

*267 patients (41%) had unknown MSI status. Abbreviations: CI, confidence interval; max, maximum; mCRC, metastatic colorectal cancer; min, minimum; MSI, microsatellite instability; MSS, microsatellite stable; OS, overall survival; PFS, progression-free survival.

**Supplementary Table 1: Data collection**

| **Types of data collected** | **Retrospective chart review** | **Prospective visits** |
| --- | --- | --- |
| **Demographic and clinical characteristics**  Date of birth, sex, race/ethnicity, socioeconomic factors  Height  Weight  Smoking status  Comorbidities (Charlson Index) | **X**  **X**  **X**  **X**  **X** | **X**  **X**  **X**  **X** |
| **Disease characteristics**  ECOG performance status  Date of diagnosis  CRC stage at diagnosis  Date of first metastases (only metachronous)  Resection of metastases (including date)  Metastases (site, number, location, synchronous/metachronous)  Sidedness of primary tumour  Molecular testing/status (RAS, BRAF, MSS/MSI phenotype)  Surgery/radiotherapy of primary tumour  CT scan, MRI, biopsy/re-biopsy | **X**  **X**  **X**  **X**  **X**  **X**  **X**  **X**  **X**  **X** | **X**  **X**  **X**  **X** |
| **mCRC treatment characteristics**  Systemic treatments received  Treatment start and end dates (or indication if ongoing)  Dose and dosing schedule  Dose adjustments and reasons for modification  Treatment discontinuation  Reasons for treatment discontinuation  Treatment setting (outpatient, hospital)  Reason for treatment initiation (patient- or tumour-related)  Surgery, radiotherapy, and other local treatment of metastases | **X**  **X**  **X**  **X**  **X**  **X** | **X**  **X**  **X**  **X**  **X**  **X**  **X**  **X**  **X** |
| **Efficacy**  Response to treatment (response, progression, stable disease)  Date of response  Method used to assess response  Date of death (if applicable) | **X**  **X**  **X** | **X**  **X**  **X**  **X** |
| **End of study**  Study end date  Reason for end of study* |  | **X**  **X** |

ACCEPT, ACCEPTance by the patient of their treatment; BFI, Brief Fatigue Inventory; CRC, colorectal cancer; CT, computerized tomography; ECOG, Eastern Cooperative Oncology Group; EORTC, European Organisation for Research and Treatment of Cancer; mCRC, metastatic colorectal cancer; MRI, magnetic resonance imaging. *Reason for end of study will be documented as loss to follow-up, study withdrawal, death, or study end date (defined as 18 months from the start of enrolment).

**Supplementary Table 2: Baseline patient characteristics by *RAS*/*BRAF* status**

| **Baseline characteristic** |  | ***RAS* mutant^#^**  **(N=361)** | ***BRAF* mutant^#^**  **(N=37)** | ***RAS*/*BRAF* wildtype**  **(N=236)** | ***RAS*/*BRAF* mutant**  **(N=9)** | **Unknown^+^**  **(N=57)** |
| --- | --- | --- | --- | --- | --- | --- |
| **Age, years*** | Median (min, max) | 68.0 (31.0, 87.0) | 65.0 (44.0, 81.0) | 65.5 (33.0, 87.0) | 68.0 (56.0, 85.0) | 70.0 (37.0, 87.0) |
| **Sex, n (%)*** | Female  Male | 164 (45.4)  197 (54.6) | 24 (64.9)  13 (35.1) | 74 (31.4)  162 (68.6) | 3 (33.3)  13 (35.1) | 19 (33.3)  38 (66.7) |
| **ECOG PS^¶^, n( %)** | 0  1  2  3  ND | 144 (41.1)  182 (52.0)  23 (6.6)  1 (0.3)  11 | 13 (37.1)  20 (57.1)  2 (5.7)  0 (0.0)  2 | 93 (39.9)  121 (51.9)  16 (6.9)  3 (1.3)  3 | 1 (11.1)  7 (77.8)  1 (11.1)  0 (0.0)  0 | 16 (29.1)  31 (56.4)  8 (14.5)  0 (0.0)  2 |
| **Time between mCRC diagnosis and PROMETCO inclusion (months)*** | Median (min, max) | 20.5 (4.9, 214.9) | 13.5 (3.4, 48.6) | 24.2 (5.6, 140.7) | 21.4 (14.2, 99.8) | 24.8 (7.5, 92.4) |
| **Number of metastatic sites, n (%)^¥^** | <3  ≥3 | 320 (88.9)  40 (11.1) | 33 (89.2)  4 (10.8) | 216 (91.5)  20 (8.5) | 9 (100.0)  0 (0.0) | 51 (89.5)  6 (10.5) |
| **Type of metastasis, n (%)*** | Synchronous  Metachronous | 243 (67.3)  118 (32.7) | 24 (64.9)  13 (35.1) | 153 (64.8)  83 (35.2) | 4 (44.4)  5 (55.6) |  |

*N=735 due to missing data; ¶Percentage based on n observed per group (i.e., not including ND values; n=716); ¥n=734 due to missing data; α, brain and skin metastases included in ‘other’; ^#^*RAS* mutant and *BRAF* mutant patient numbers excludes *RAS/BRAF* mutant patients; ^+^Unknown only includes patients with unknown *BRAF* and *RAS* status (excludes patients with *BRAF* wildtype/unknown *RAS* status [n=6], and patients with *RAS* wildtype/unknown *BRAF* status [n=29]); Abbreviations: ECOG PS, Eastern Cooperative Oncology Group performance status; max, maximum; mCRC, metastatic colorectal cancer; min, minimum; MSI, microsatellite instability; MSS, microsatellite stable; mut, mutant; ND, not determined; WT, wild-type.

**Supplementary Table 3: Baseline patient characteristics by MSI status***

| **Baseline characteristic** |  | **MSI-high**  **(N=12)** | **MSS**  **(N=430)** | **Unknown**  **(N=295)** |
| --- | --- | --- | --- | --- |
| **Age, years** | Median (min, max) | 60 (31, 77) | 67 (59, 74) | 68 (61, 74) |
| **Sex, n (%)** | Female  Male | 4 (33.3)  8 (67.7) | 174 (43.0)  231 (57.0) | 166 (56.8)  126 (43.2) |
| **ECOG PS^¶^, n (%)** | 0  1  2  3  ND | 4 (36.4)  6 (54.5)  1 (9.1)  0 (0.0)  1 (9.1) | 149 (38.0)  206 (52.6)  35 (8.9)  2 (0.5)  0 (0.0) | 116 (40.4)  155 (54.0)  15 (5.2)  1 (0.3)  8 (2.8) |
| **Time between mCRC diagnosis and PROMETCO inclusion (months)** | Median (min, max) | 24.1 (7.1, 44.6) | 14.6 (4.9, 214.9) | 16.9 (3.4, 114.1) |
| **Number of metastatic sites, n (%)^¥^** | <3  ≥3 | 12 (100.0)  0 (0.0) | 362 (89.4)  43 (10.6) | 262 (90.0)  29 (10.0) |
| **Type of metastasis, n (%)** | Synchronous  Metachronous | 7 (58.3)  5 (41.7) | 267 (65.9)  138 (34.1) | 187 (64.0)  105 (36.0) |

*N=734 due to missing data; ¶Percentage based on n observed per group (i.e., not including ND values; n=716); ¥n=734 due to missing data; α, brain and skin metastases included in ‘other’; Abbreviations: ECOG PS, Eastern Cooperative Oncology Group performance status; max, maximum; mCRC, metastatic colorectal cancer; min, minimum; MSI, microsatellite instability; MSS, microsatellite stable; mut, mutant; ND, not determined; WT, wild-type.

**Supplementary Table 4: mCRC treatments* by BRAF/RAS mutation status**^¶^

| **Treatment, n (%)^¥^** | ***RAS* mutant**  **(N=361)** | ***BRAF* mutant**  **(N=37)** | ***RAS*/*BRAF* wildtype**  **(N=235)** | ***RAS*/*BRAF* mutant**  **(N=9)** | **Unknown**  **(N=92)** |
| --- | --- | --- | --- | --- | --- |
| Fluoropyrimidine (5-FU or capecitabine or tegafur) | 358 (99.2) | 37 (100.0) | 233 (99.1) | 9 (100.0) | 90(97.8) |
| Oxaliplatin | 330 (91.4) | 31 (83.8) | 202 (86.0) | 7 (77.8) | 79 (85.9) |
| Irinotecan | 349 (96.7) | 34 (91.9) | 228 (97.0) | 9 (100.0) | 86 (93.5) |
| Bevacizumab | 298 (82.5) | 27 (73.0) | 142 (60.4) | 9 (100.0) | 64 (69.6) |
| Aflibercept | 93 (25.8) | 9 (24.3) | 34 (14.5) | 3 (33.3) | 9 (9.8) |
| Ramucirumab | 4 (1.1) | – | 3 (1.3) | 1 (11.1) | 1 (1.1) |
| Anti-VEGF (bevacizumab or aflibercept or ramucirumab) | 305 (84.5) | 31 (83.8) | 167 (71.1) | 9 (100.0) | 66 (71.7) |
| Anti-EGFR (panitumumab or cetuximab) | 7 (1.9) | 27 (73.0) | 212 (90.2) | 2 (22.2) | 46 (50.0) |
| Pembrolizumab | – | – | 2 (0.9) | – | – |
| Nivolumab | 4 (1.1) | – | 2 (0.9) | – | 1 (1.1) |
| Avelumab | – | – | – | – | 1 (1.1) |
| Atezolizumab | 1 (0.3) | – | – | – | – |
| Encorafenib + cetuximab | – | 17 (45.9) | – | 1 (11.1) | 1 (1.1) |
| FTD/TPI | 292 (80.9) | 18 (48.6) | 166 (70.6) | 9 (100.0) | 67 (72.8) |
| Regorafenib | 111 (30.7) | 10 (27.0) | 48 (20.4) | 1 (11.1) | 16 (17.4) |

*Administered from mCRC diagnosis to death; ^¶^N=734 due to missing data; **^¥^**At least one treatment. Abbreviations: 5-FU, 5-fluorouracil; EGFR, epidermal growth factor receptor; FTD/TPI, trifluridine/tipiracil; mCRC, metastatic colorectal cancer; VEGF, vascular endothelial growth factor.
